# Oxidative Desulfurization of Fuel Oil by Pyridinium-Based Ionic Liquids

**DOI:** 10.3390/molecules14114351

**Published:** 2009-10-28

**Authors:** Dishun Zhao, Yanan Wang, Erhong Duan

**Affiliations:** 1 School of Chemical Engineering, Tianjin University, Tianjin, 300072, China; E-Mail: mmsafin@hotmail.com; 2 School of Chemistry and Pharmaceutical Engineering, Hebei University of Science and Technology, Shi jiazhuang 050018, China; 3 School of Environmental Science and Engineering, Hebei University of Science and Technology, Shijiazhuang 050018, China; E-Mail: deh@tju.edu.cn

**Keywords:** ionic liquids, extraction-oxidation, desulfurization, pyridinium-based

## Abstract

In this work, an *N*-butyl-pyridinium-based ionic liquid [BPy]BF_4_ was prepared. The effect of extraction desulfurization on model oil with thiophene and dibenzothiophene (DBT) was investigated. Ionic liquids and hydrogen peroxide (30%) were tested in extraction-oxidation desulfurization of model oil. The results show that the ionic liquid [BPy]BF_4_ has a better desulfurization effect. The best technological conditions are: V(IL)/V(Oil) /V(H_2_O_2_) = 1:1:0.4, temperature 55 °C, the time 30 min. The ratio of desulfurization to thiophene and DBT reached 78.5% and 84.3% respectively, which is much higher than extraction desulfurization with simple ionic liquids. Under these conditions, the effect of desulfurization on gasoline was also investigated. The used ionic liquids can be recycled up to four times after regeneration.

## 1. Introduction

The dramatic environmental impact of sulfur oxides contained in engine exhaust emissions has been widely recognized in recent years. As a result specifications regarding sulfur content in fuels are becoming more and more stringent [1], and desulfurization of gasoline has attracted wide attention. In European countries, the sulfur component in gasoline was restricted to a maximum 50 μg/mL in 2005, and gasoline will be sulfur-free in 2011 [2]. Research on deep desulfurization of gasoline is of great importance in both academic and industrial fields.

A traditional desulfurization method is catalytic hydrodesulfurization (HDS) using catalysts such as CoMo or NiMo [3], which requires both high temperature and high pressure. Also HDS is only effective on aliphatic sulfur structures such as thiols, thioethers and disulfides, etc. Sulfur-containing aromatic compounds (including thiophene and its derivatives) are barely removed by this process. Due to this reason, alternative desulfurization technologies have been developed, including adsorption [4], extraction [5], and selective oxidation [6]. Extractive desulfurization is believed to be one of the best methods because the process operation is easy. However, using volatile organic compounds (VOCs) as extractant leads to more environmental and safety concerns.

Ionic liquids (ILs) have been employed extensively in Green Chemistry. ILs have the characteristics of nonvolatility, nonflammablity and high thermal stability. As a result, ILs are considered as green solvents and are employed in processes of catalysis, chemical synthesis and separations. The use of ILs as extractants to remove aromatic sulfur compounds in extractive desulfurization has been reported in recent research studies [7,8,9,10,11,12,13,14]. Bösmann [7] first described that imidazolium-based ILs can be utilized as extractant. Most of the literatures have described with ILs as simple extractant; the results indicate that ionic liquids can efficaciously extract the sulfur content from the oil phase. Wen-Hen Lo and co-workers [8] combined the methods of oxidation and extraction, using, H_2_O_2_ - acetic acid as the oxidant, and [BMIM]BF_4_ and [BMIM]PF_6_ as the extractant; this process increases the desulfurization rate by about an order of magnitude relative to that of merely extracting with RTILs.

According to the reports [14], the presence of acid could increase the desulfurization, but when it comes to industrial applications, the equipment would be corroded by acid. In this work, we use a type of pyridinium-based IL [BPy]BF_4_ as extractant, which is combined with hydrogen peroxide as oxidant for the first time. The desulfurization of thiophene and dibenzothiophene (DBT) in model oil as well as fluidized catalytic cracking (FCC) gasoline are studied.

## 2. Results and Discussion

Both the cation of IL and thiophene (as well as its derivatives) have aromatic rings. When the IL cation, which had an aromatic ring structure with a large π bond, interacted with thiophene sulfur compounds, a complex was produced and the thiophene compounds were extracted to the IL phase due to the easily polarized sulfur molecule [15]. 

### 2.1. Effect of extractive desulfurization for model oil

**Figure 1:**
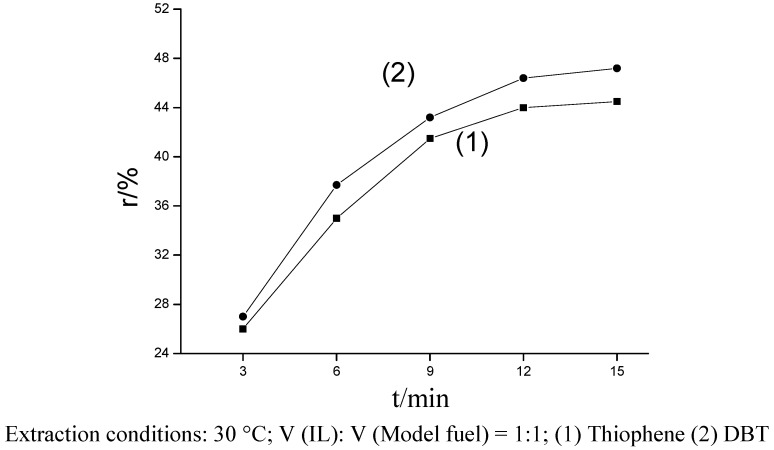
Effect of desulfurization to thiophene and DBT in model fuel with IL.

The extractive desulfurization results of IL[BPy]BF_4_ are shown in Figure 1. When IL was used as extractant for thiophene and DBT containing model oil, the extraction equilibrium will reached after about 15 min. the ratio of sulfur removal reached 44.5% and 47.2%, respectively, with the reaction conditions set at 30 °C for 15 min. The IL has better extracting ability to remove DBT than thiophene from the model oil, the reason may be that DBT has more aromatic rings on the thiophene branch, so the interaction effect between them was enhanced.

### 2.2. Effects of oxidative desulfurization for model oil

**Figure 2:**
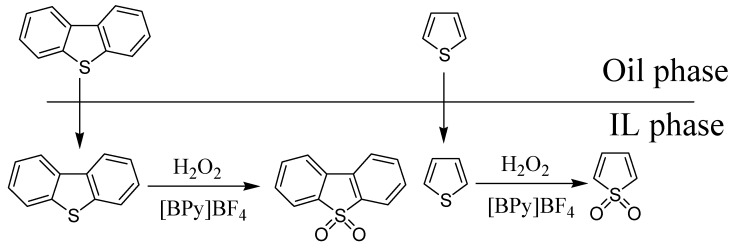
Process of extraction-oxidation of thiophene and DBT in model oil by IL- H_2_O_2_ system.

Because hydrogen peroxide can be dissolved in the IL, upon addition of hydrogen peroxide as an oxidant into IL[BPy]BF_4_, the H_2_O_2_ was decomposed to hydroxyl radical in the system, and then was further decomposed to hydroxyl radical (HOO-). This oxidizes the thiophene sulfides to the corresponding sulfone, and as the polarity of the sulfone was larger, it was remained in the IL phase. The process of this reaction is shown in Figure 2.

#### 2.2.1. Effect of the amount of H_2_O_2_ on desulfurization of model oil

**Figure 3:**
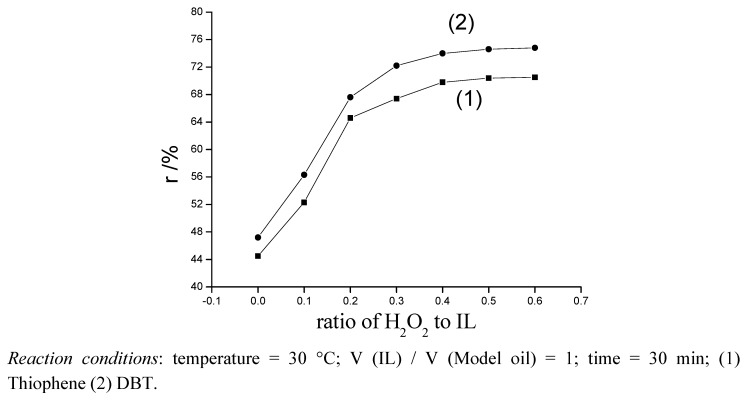
Effect of amount of H_2_O_2_ on desulfurization in model oil.

The influence of the amount of H_2_O_2_ (30%) on desulfurization is shown in Figure 3. The rate of desulfurization was not changed after about 30 min. It is obvious that the desulfurization of model oil is increased after adding H_2_O_2_ to IL, with the presence of H_2_O_2_, the desulfurization were up from 44.5% to 69.8% (thiophene) and from 47.2% to 74.0% (DBT). The optimal ratio by volume of H_2_O_2_ to IL is 0.4:1. No change in the desulfurization was visible when the ratio was greater. This means that when the ratio is 0.4:1, free radicals decomposed by H_2_O_2_ were sufficient to oxidize thiophene and DBT to sulfone, so we chose the ratio of 0.4:1 in the subsequent research.

#### 2.2.2. Effect of temperature on desulfurization of model oil

**Figure 4:**
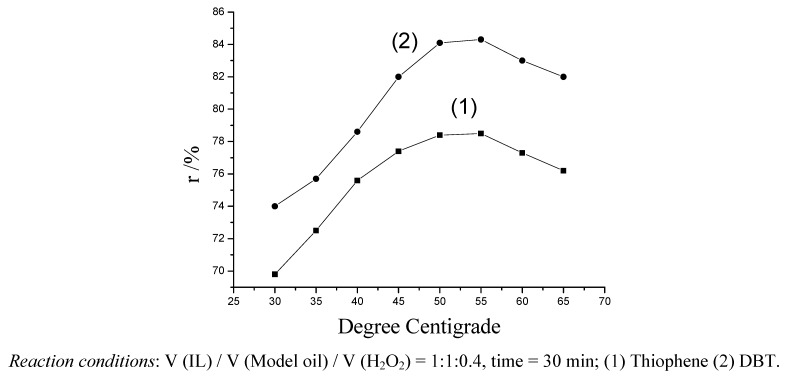
Effect of reaction temperature on desulfurization in model oil.

The effect of reaction temperature (from 30 °C to 65 °C) on the desulfurization of model oil is shown in Figure 4. The reaction rate increased obviously when the temperature increased from 30 °C to 55 °C, and when the temperature increased to 55 °C, the desulfurization reached the maximum 78.5% and 84.3% values, respectively. When the temperature was higher than 55 °C, the desulfurization decreased appreciably. The reason is that the H_2_O_2_ is decomposed to H_2_O at a high temperature. The temperature of 55 °C was the optimal one in this study.

### 2.3. Oxidative desulfurization of gasoline with IL

The extraction system and extraction-oxidation system were applied to gasoline containing 780 μg/mL sulfur, which was provided by the Shijiazhuang Refinery. The reaction conditions for the extraction desulfurization ares: mass ratio of IL to gasoline is 1:1, reaction for 30 min at 55 °C. The reaction conditions of extraction-oxidation desulfurization are: mass ratio of IL to gasoline is 1:1, the amount of H_2_O_2_ to IL is 0.4:1, time and temperature are the same. The concentrations of sulfur in gasoline before and after reaction are shown in Table 1, where it can be seen that the sulfur content of gasoline can be reduced by 35.9% (extraction desulfurization) and 56.3% (extraction-oxidation desulfurization). We found that the desulfurization was decreased compared to the data of desulfurization on model oil. The reason is that gasoline contains many non-aromatic sulfur components such as thiol sulfide which cannot be extracted by IL effectively. On the other hand, the aromatic hydrocarbons and olefins in gasoline influence the sulfur extraction as well.

**Table 1:** Effect on desulfurization in diesel oil.

IL system	Primal sulfur content	Desulfurization
Extraction^a^	780μg/mL	35.9%
Extraction-oxidation^b^	780μg/mL	56.3%

*Reaction conditions*: a: temperature = 55 °C; V (Model oil) / V (IL) = 1:1; time = 30 min; b: temperature = 55 °C; V (Model oil) / V ( IL) / V(H_2_O_2_) = 1:1:0.4; time = 30 min.

### 2.4. Regeneration of used IL

The recycling of the xtraction-oxidation system for IL was investigated in the desulfurization of model oil with thiophene and DBT. The used IL was distilled in an oil bath at 110 °C to remove H_2_O_2_ entirely, and then the IL was regenerated by re-extraction in tetrachloromethane. The results of desulfurization on model oil by regenerated IL are shown in Table 2, which indicates that the regenerated IL could be recycled four times with negligible decrease in activity.

**Table 2:** The desulfurization of model oil by regenerated IL.

Regenerated time	Thiophene	DBT
Fresh IL	78.5%	84.3%
1	77.0%	83.2%
2	76.4%	82.0%
3	75.8%	81.1%
4	74.3%	80.3%
5	72.1%	77.5%

*Reaction conditions*: temperature = 55 °C; V (Model oil) / V (IL) / V (H_2_O_2_) = 1:1:0.4; time = 30 min.

## 3. Experimental

The IL [BPy]BF_4 _was prepared using the methods described in the literature [16]. Briefly, pyridine (0.2 mol), butyl bromide (0.2 mol) and cyclohexane (50 mL) were stirred in a round-bottom flask at 60 °C for 12 h. The white precipitate (*N*-butylpyridinium bromide) was filtrated off and evaporated in a vacuum drying oven. The IL was prepared by mixing *N*-butylpyridinium bromide (0.2 mol) and sodium tetrafluoroborate (0.2 mol) with acetone (100 mL) in a round-bottom flask, and stirring the mixture at room temperature for 12 h. The precipitate was filtered off, and then the solvent was removed by rotary evaporation to leave the desired yellow IL. The IL was analyzed by ^1^H-NMR, ^13^C-NMR, and Fourier Transform Infrared (FTIR) spectroscopy.

Model oil was prepared by dissolving thiophene (0.525 g) in *n*-octane (500 mL), and DBT (0.920 g) in *n*-octane (500 mL), The sulfur contents of the model oils were 400 μg/mL and 320 μg/mL respectively. The gasoline was provided by the Shijiazhuang Refinery of China, The sulfur content was 780 μg/mL. Desulfurization experiments were carried out as follows: model oil (or gasoline) (10 mL)e and IL (10 mL) and a certain amount of hydrogen peroxide were added to a 50 mL flask, the resulting mixture was stirred for 30 min at the desired reaction temperature. The concentration of sulfur in model oil and FCC gasoline were detected by a microcoulometric detector (WK-2D) and a gas chromatograph equipped with a flame photometric detector (GC-FPD).

## 4. Conclusions

The ionic liquid [BPy]BF_4_ was found to be effective in the extraction of sulfur components in model oils and gasoline at room temperature. The desulfurization process was achieved after H_2_O_2 _was added to the extraction system; the oxidizability of H_2_O_2_ was utilized for oxidizing the sulfur components to the corresponding sulfones which were extracted by the IL. The desulfurization ofthiophene and DBT by the extraction-oxidation system can reach values of 78.5% and 84.3%, respectively, which are much higher than those achieved by extractive desulfurization. Because of the other complex components in gasoline, the desulfurization of that substance was lower than the desulfurization of model oil. The used IL was regenerated through re-extraction in tetrachloro-methane, and can be recycled four times without any obvious decrease in activity. This proves the feasibility of desulfurization with a simple, mild and environmentally benign method in industry in the future.
